# Effects of iron chelation therapy on the clinical course of aceruloplasminemia: an analysis of aggregated case reports

**DOI:** 10.1186/s13023-020-01385-w

**Published:** 2020-04-25

**Authors:** Lena H. P. Vroegindeweij, Agnita J. W. Boon, J. H. Paul Wilson, Janneke G. Langendonk

**Affiliations:** 1 Department of Internal Medicine, Center for Lysosomal and Metabolic Diseases, Porphyria Center Rotterdam, Erasmus MC University Medical Center Rotterdam, Rotterdam, The Netherlands; 2 Department of Neurology, Erasmus MC University Medical Center, Rotterdam, The Netherlands

**Keywords:** Aceruloplasminemia, Neurological manifestations, Iron chelation therapy, Deferiprone, Phlebotomy

## Abstract

**Background:**

Aceruloplasminemia is a rare genetic iron overload disorder, characterized by progressive neurological manifestations. The effects of iron chelation on neurological outcomes have only been described in case studies, and are inconsistent. Aggregated case reports were analyzed to help delineate the disease-modifying potential of treatment.

**Methods:**

Data on clinical manifestations, treatment and neurological outcomes of treatment were collected from three neurologically symptomatic Dutch patients, who received deferiprone with phlebotomy as a new therapeutic approach, and combined with other published cases. Neurological outcomes of treatment were compared between patients starting treatment when neurologically symptomatic and patients without neurological manifestations.

**Results:**

Therapeutic approaches for aceruloplasminemia have been described in 48 patients worldwide, including our three patients. Initiation of treatment in a presymptomatic stage of the disease delayed the estimated onset of neurological manifestations by 10 years (median age 61 years, SE 5.0 vs. median age 51 years, SE 0.6, *p* = 0.001). Although in 11/20 neurologically symptomatic patients neurological manifestations remained stable or improved during treatment, these patients were treated significantly shorter than patients who deteriorated neurologically (median 6 months vs. median 43 months, *p* = 0.016). Combined iron chelation therapy with deferiprone and phlebotomy for up to 34 months could be safely performed in our patients without symptomatic anemia (2/3), but did not prevent further neurological deterioration.

**Conclusions:**

Early initiation of iron chelation therapy seems to postpone the onset of neurological manifestations in aceruloplasminemia. Publication bias and significant differences in duration of treatment should be considered when interpreting reported treatment outcomes in neurologically symptomatic patients. Based on theoretical grounds and the observed long-term safety and tolerability in our study, we recommend iron chelation therapy with deferiprone in combination with phlebotomy for aceruloplasminemia patients without symptomatic anemia.

## Background

Aceruloplasminemia (OMIM #604290) is a rare form of neurodegeneration with brain iron accumulation (NBIA), characterized by progressive neurological deterioration and iron accumulation in visceral organs [1]. Iron chelation therapy has been shown by biochemical markers and quantitative visceral MRI to reduce body iron stores, but the reported effects on neurological outcomes are inconsistent [2, 3].

Iron accumulation in aceruloplasminemia is caused by a lack of ceruloplasmin ferroxidase activity. The failure to convert ferrous iron (Fe^2+^) to ferric iron (Fe^3+^) at the cell surface results in low transferrin saturation [1, 4, 5]. Deferiprone is the only chelator available capable of transporting iron across cell membranes and across the blood-brain barrier. At low transferrin saturation, deferiprone additionally mediates shuttling of iron from excess iron stores to erythroid cells by transferring iron to transferrin [6]. However, deferiprone monotherapy is not highly effective in reducing body iron stores in aceruloplasminemia [7, 8], and needs to be combined with another chelator to achieve satisfactory rates of iron removal [8–10]. The deferiprone-induced redistribution of iron to hematopoietic tissues might permit a combination with phlebotomy as a new therapeutic approach for aceruloplasminemia. Deferiprone with phlebotomy has the potential to reduce both cerebral and systemic iron stores more rapidly than previously achieved, and possibly to slow disease progression.

Due to the rarity of aceruloplasminemia, randomized controlled study designs are not feasible to explore clinical outcomes of iron chelation therapy. The value of aggregated case reports has recently been highlighted as an alternative means of systematically deriving evidence from the literature in rare diseases [11, 12]. However, the rich source of information embedded in published case reports and case series has not yet been quantitatively investigated in such manner for aceruloplasminemia.

We performed an analysis of aggregated case reports of aceruloplasminemia and present detailed clinical outcomes of long-term treatment with deferiprone and phlebotomy in three Dutch patients. The aim of our study was to increase knowledge of the natural history of aceruloplasminemia and disease-modifying potential of iron chelation therapy.

## Methods

### Subjects

Three Dutch patients with homozygosity for G631R in the ceruloplasmin (*CP)* gene were enrolled in the study: two brothers with neurological symptoms and their first cousin with minimal neurological complaints. The neurological presentation of all patients has been previously published [13–16]. The study was approved by the Medical Ethics Review Board of the Erasmus MC. All participants gave written informed consent.

### Procedures

Deferiprone was combined with phlebotomy in all patients. Deferiprone starting dose was 15 mg/kg/day given orally in two doses, followed by titration to a maximum dose of 65 mg/kg/day. Phlebotomy was started by withdrawing 300 ml blood every fortnight and subsequently increased to 500 ml every 3 weeks.

Safety and tolerability of deferiprone were strictly monitored, as deferiprone is rarely associated with neutropenia (neutrophil count < 1.5·10^9^/l) and agranulocytosis (neutrophil count < 0.5·10^9^/l). Neutrophils were measured weekly during deferiprone titration and every 2 weeks after the maximum dose of deferiprone had been reached. In patients who developed neutropenia, the dose of deferiprone was halved and neutrophil counts were monitored weekly. If neutrophil counts recovered, deferiprone was gradually increased to its original dosage. If neutrophils remained below the lower limit, deferiprone was stopped and not reintroduced. Transaminases (AST, ALT) were measured monthly and zinc levels every 6 months. Hemoglobin levels were measured prior to each phlebotomy. Phlebotomy was postponed if the hemoglobin level was more than 3.25 g/dl lower (2 mmol/l) than at baseline or the patient mentioned complaints associated with anemia.

Effectiveness was assessed every 3 months during the first year, followed by half-year evaluations in the following years of treatment. Neurological function was evaluated and videotaped by a movement disorders specialist (A.B.). The Unified Parkinson’s Disease Rating Scale *(UPDRS/III)* was used to measure motor symptoms of parkinsonism and the Scale for the Assessment and Rating of Ataxia *(SARA)* for the assessment of coordination in several tasks including speech and gait. The Hospital Anxiety and Depression Scale *(HADS)* evaluated levels of anxiety and depression and was used as a self-assessment questionnaire. Ophthalmic examinations for the assessment of retinopathy were performed at yearly intervals. Iron depositions in the brain were qualitatively assessed by yearly T2 and T2*-weighted MRI, as hypo-intensities on these sequences are indicative of iron accumulation. Images were acquired using an individualized protocol at either 1.5 T or 3 T, depending on the magnetic field strength of the first MRI, and evaluated by a neuroradiologist. Quantitative comparisons could not be performed. Systemic iron stores were followed by serum ferritin and HbA1c levels. Serum ferritin, reflecting iron stores in the liver, was determined together with CRP, AST and ALT, to permit interpretation of changes in serum ferritin values. HbA1c was used as a marker of glycemic control and development of diabetes following pancreatic iron accumulation.

### Treatment parameters

The primary endpoints were safety and change from baseline in *UPDRS/III* and *SARA* scores. Changes in *HADS* scores, qualitative assessment of brain iron as evaluated by MRI, and changes in ferritin and HbA1c values served as secondary effectiveness endpoints.

### Review of the literature

Case reports and case series that described treatment for aceruloplasminemia were collected by updating a previously published literature search till December 2019 [13]. Gender, ethnicity, age at first manifestations, age at onset of neurological manifestations, age at initiation of treatment, duration of treatment, neurological outcomes of treatment and age at death were extracted from all identified case studies. The authors of these cases were asked for additional follow-up of the patients.

### Statistics

Ages and duration of treatment were presented as median (interquartile range) values. Differences in gender, ethnicity, age at first manifestations, age at initiation of treatment and duration of treatment between patients with neurological manifestations at the start of treatment and neurologically asymptomatic patients were explored using the Mann-Whitney U-test or χ^2^ test, where appropriate. Differences in the clinical course of the disease and onset of neurological manifestations were explored using Kaplan-Meier analysis. Patients who remained neurologically asymptomatic were censored at the age at end of follow-up, which was obtained from either later published articles or direct contact with the authors. If additional follow-up was not available, the age at end of follow-up was calculated using the reported age at initiation of treatment and duration of treatment. Statistical significance was defined as *p* < 0.05. All analyses were performed using SPSS 25 (IBM, Armonk, NY).

## Results

### Deferiprone with phlebotomy in three G631R homozygous patients

At the time of enrolment, both brothers (case 1, 2) had a two-year history of neurological manifestations and had been treated with phlebotomy monotherapy for several months, which resulted in mildly reduced hemoglobin levels. Both presented initially with normal hemoglobin levels. Their first cousin (case 3) reported stable neurological disease after 13 years of deferoxamine treatment (1000 mg/day, s.c., twice weekly) [16]. He was known with microcytic anemia since childhood, which had been treated intermittently with oral iron supplements. At the time of counselling, microcytic anemia was considered to be the first manifestation of aceruloplasminemia in case 3, following routine exclusion of other causes.

The combination of deferiprone and phlebotomy was administered for 15 months in case 1, 34 months in case 2 and 1 month in case 3. Total patient follow-up ranged from 18 to 76 months. Premature discontinuation of treatment was due to neurological deterioration in case 1 and 2, in whom the combination of deferiprone and phlebotomy was generally well tolerated. In case 3, phlebotomy was discontinued due to anemia-related fatigue and dyspnea following two venesections of 300 ml. He was subsequently treated by deferiprone in combination with deferoxamine (1000 mg/day s.c., once-twice weekly). A detailed overview of the biochemical sequelae and tolerance of iron chelation therapy in our three cases can be obtained from Additional file 1.

**Fig. 1:**
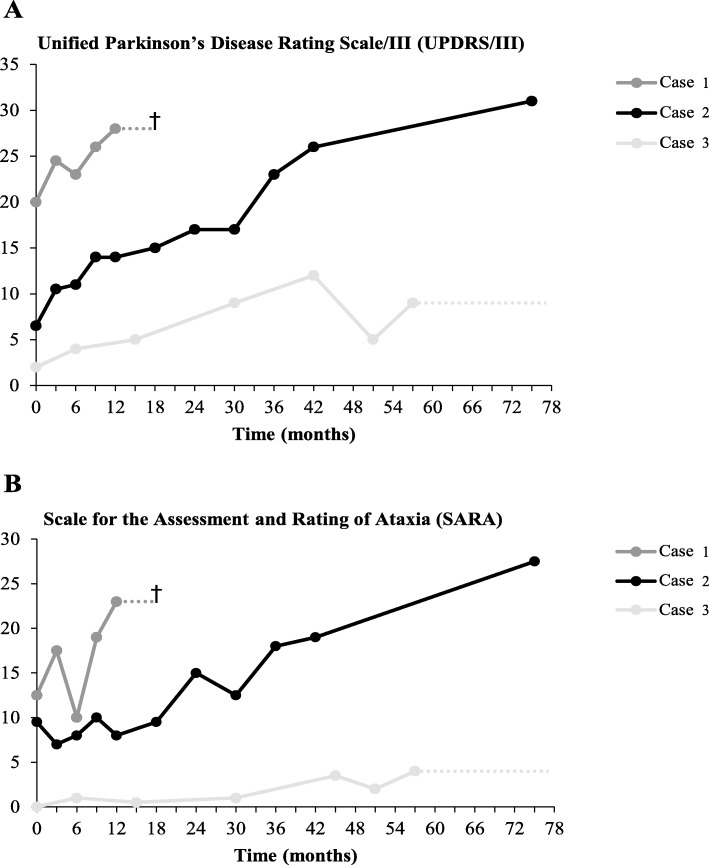
Neurological rating scales of the G631R homozygous cases at baseline and during follow-up. Dotted lines indicate that motor function tests were no longer performed, which was due to neurological deterioration in case 1 and hospital relocation in case 3

Table 1 summarizes the clinical outcomes of treatment with deferiprone and phlebotomy (case 1, 2) and deferiprone in combination with deferoxamine (case 3). All patients deteriorated neurologically, as illustrated by the gradual increase in *UPDRS/III* and *SARA* scores over time (Fig. 1). Patient ratings of anxious and depressive feelings *(HADS)* had only been consistently collected in case 1 and 2*,* and were highly variable during treatment. Diabetes became manifest despite treatment in case 1 and 3, while in case 2, who was already known with insulin-dependent diabetes at baseline, glucose and HbA1c levels improved and insulin doses could be reduced.

**Table 1:** Clinical characteristics of our G631R homozygous patients at baseline and end of follow-up

Case/gender/age^a^	Follow-up (months)	Neurological function		Retinopathy		Diabetes		Anemia	
		Baseline	EFU	Baseline	EFU	Baseline	EFU	Baseline	EFU
1/M/50	18	Orofacial dyskinesia, chorea, dystonia, dysarthria, gait disturbance	Died	No	NA	No	Yes	Yes^b^	Yes
2/M/56	76	Dysarthria, ataxia, gait disturbance, behavioral changes	Progressive speech and gait disturbance, wheelchair-bound, aspiration, cognitive decline and apathy	No	No	Yes	Yes	Yes^b^	No
3/M/61	70	Stress related tremor, slightly diminished facial expression	Development of gait disturbance with falls, mental slowing, behavioral changes	No	No	No	Yes	Yes	Yes

End of follow-up data (EFU) of case 3 are based on inquiries. Other abbreviations: NA- not available

^a^Age at baseline. ^b^Case 1 and 2 initially presented with normal hemoglobin levels; previous treatment with phlebotomy monotherapy had resulted in mild anemia

T2- and T2*-weighted MRI showed excessive iron accumulation in all cases at baseline, predominantly affecting basal ganglia, red nucleus, dentate nucleus and thalamus. Imaging was performed at 1.5 T in case 1 and 3 T in case 2 and 3. Qualitative follow-up revealed no significant changes in the pattern of iron accumulation nor in the severity of hypointensities in case 2 and 3, while in case 1 progressive darkening of deep grey matter regions was observed (Additional file 2).

### Review of the literature

Therapeutic approaches for aceruloplasminemia have been described in 48 cases worldwide [7, 8, 15–49], including our three patients. Overall, 13 different iron chelating methods have been administered, of which a detailed overview is included in Additional file 3. Monotherapy with deferoxamine or deferasirox has been most frequently reported, while combined iron chelation therapy was limited to 5 previously reported cases [8, 17, 40, 44, 48]. In these patients, phlebotomy was combined with deferasirox, deferiprone with deferoxamine, and fresh frozen plasma with deferoxamine or deferiprone. In 7 others, an iron chelator was combined with zinc, vitamin C or E [17, 30, 33]. The consecutive use of different chelators or combinations of iron chelating methods has been described in 10 patients [7, 16–19, 21, 22, 24, 27].

Table 2 summarizes the clinical characteristics of all 48 cases treated for aceruloplasminemia, differentiated between neurologically symptomatic patients (*n* = 24) and patients who started treatment before the onset of neurological manifestations (*n* = 24). These neurologically asymptomatic patients were diagnosed following the onset of diabetes and/or anemia [7, 17, 22, 28, 43, 47], during the diagnostic workup of unexplained hyperferritinemia with low transferrin saturation [8, 17, 20, 23, 39, 43, 46, 47], or by family counselling [16, 21, 24, 25, 27, 31, 37, 42].

**Table 2:** Clinical characteristics of patients with and without neurological manifestations when treatment was initiated

	Symptomatic *n* = 24		Asymptomatic *n* = 24		*p*
Male gender (%)	11 (45.8)		13 (54.2)		0.564
Japanese ethnicity (%)	8 (33.3)		4 (16.7)		0.182
*Natural history*					
Age at first manifestations, y	35 (26–49)		36.5 (20–43)		0.443
Age at first neurological manifestations, y	51 (48.5–55)		–		**–**
*Treatment and clinical outcomes*					
Age at start of treatment, y	55 (50.5–59)		39.5 (35–49)		0.001
Duration of treatment, m	14 (5.5–48)		72 (24–120)		0.001
Age at end of follow-up, y	58 (54–63)		53 (47–61)		0.08
Age at first neurological manifestations, y	–		50 (45–55)	*n* = 9	**–**
Age at death, y	60 (56–65.5)	*n* = 8	67	*n* = 1	–

Abbreviations: *n* number, *y* years, *m* months

Ages and duration of treatment are expressed as median (interquartile range)

**Fig. 2:**
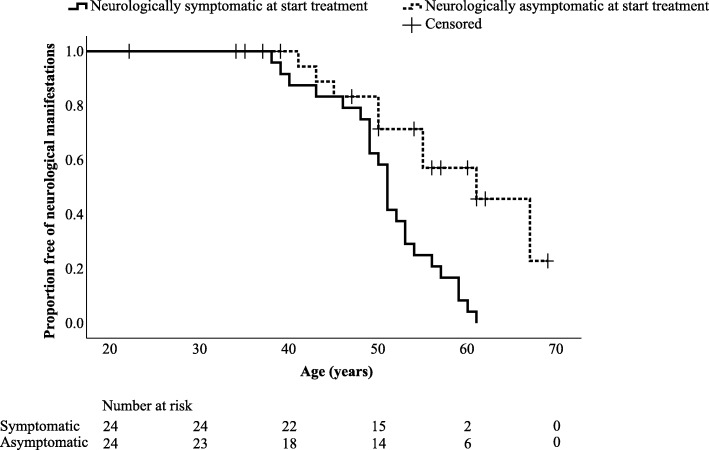
Onset of neurological manifestations during treatment for aceruloplasminemia compared with natural course of the disease

Treatment in neurologically asymptomatic patients was started 11.5 years before neurological sequelae of aceruloplasminemia were likely to develop, based on the natural course of the disease observed in neurologically symptomatic patients (*p* = 0.003). However, the age at end of follow-up of initially asymptomatic patients was not significantly different from the age at onset of neurological manifestations in the symptomatic group (*p* = 0.918), while only 9 of the 24 initially asymptomatic patients had developed neurological manifestations at that age. Kaplan-Meier analysis revealed that initiation of treatment in a presymptomatic stage of the disease might delay the onset of neurological manifestation by 10 years (*p* = 0.001, Table 3 and Fig. 2).

**Table 3:** Onset of neurological manifestations stratified by timing of treatment initiation

Group	Median age at first neurological manifestations (years)	SE	95% CI	*p*
Symptomatic at start treatment	51	0.6	49.8–52.2	0.001
Asymptomatic at start treatment	61	5.0	51.2–70.8

Clinical outcomes of treatment in neurologically symptomatic patients were variable. Overall, neurological manifestations remained stable or improved in 11 patients, while 9 patients deteriorated neurologically despite treatment. In 4 cases, clinical outcomes of treatment were not available. Quantitative evaluations of neurological function were available in 8 of the 48 cases, using various clinical rating scales. More detailed information on the neurological outcomes of treatment in individual patients can be obtained from Additional file 3. It should be noted, though, that the median duration of treatment in patients who remained stable or improved neurologically was significantly shorter than patients in whom neurological deterioration was observed (6 months vs. 43 months, *p* = 0.016). The limited available data on age at death precluded further analysis of any treatment-related influences on mortality in aceruloplasminemia.

## Discussion

Data from published case reports and case series of aceruloplasminemia were combined to improve our understanding of the natural history of the disease and to systematically address the disease-modifying potential of iron chelation therapy. In addition, this is the first study that describes combined iron chelation therapy with deferiprone and phlebotomy for aceruloplasminemia.

Results of up to 3 years of combined iron chelation therapy with deferiprone and phlebotomy showed that the combination can be safely performed in aceruloplasminemia patients when treatment is subject to strict monitoring of hemoglobin and granulocyte count. This new therapeutic approach was initiated in three Dutch patients at a neurologically symptomatic stage of the disease. One patient, who was known with microcytic anemia, did not tolerate phlebotomy and was subsequently treated by deferiprone in combination with deferoxamine. Although neither deferiprone with phlebotomy nor deferiprone with deferoxamine could prevent further neurological deterioration, this report yields detailed information on clinical manifestations, adverse events associated with treatment and disease progression in various stages of the disease, which might be valuable for individual patient management.

The rate of neurological deterioration was markedly different between our patients. The slower progression of neurological manifestations in case 3, who had minimal neurological complaints at baseline, might emphasize the importance of early initiation of iron chelation therapy to provide clinical benefit. The analysis of aggregated case reports of aceruloplasminemia supports this interpretation, as the estimated onset of neurological manifestations could be postponed by 10 years when treatment was initiated in a presymptomatic stage of the disease. In a recently published randomized controlled trial in patients with pantothenate kinase-associated neurodegeneration (PKAN), the most common form of NBIA, deferiprone seemed to have more potential to slow disease progression when it was initiated in patients with less advanced stages of neurodegeneration [50]. As such analyses of the rate of neurological progression and disease-modifying potential of treatment require quantitative evaluations of neurological function, accepted clinical rating scores should ideally be included in future case studies of aceruloplasminemia.

One of the major pitfalls of using case reports and case series is publication bias – cases with a positive response to treatment are much more likely to be published than those without clinical benefit [51, 52]. This could account for the discrepancy between the progression of neurological manifestations in our patients and the relatively high proportion of published cases that showed improvement of neurological sequelae during follow-up. The majority of these neurologically symptomatic cases that improved were followed over months, while results of those with long-term follow-up illustrated the debilitating nature of aceruloplasminemia and were in accordance with observations in our patients.

Treatment outcomes in neurologically asymptomatic patients are subject to specific types of information bias: lead-time bias and length-time bias. Lead-time bias indicates the overestimation of survival duration due to detection of disease in an early stage, while length-time bias addresses the systematic error resulting from a relative excess of cases with a slower rate of disease progression [53–55]. Although both types of information bias have been predominantly discussed in relation to cancer screening, lead-time bias should be considered when interpreting the sustained absence of neurological manifestations in reported aceruloplasminemia patients in whom treatment was initiated years before neurological manifestations were to expect. On the other hand, nearly half of the patients in whom treatment was initiated in a presymptomatic stage of the disease developed neurological manifestations above 55 years of age or remained asymptomatic despite being in their late fifties or sixties. Length-time bias might have additionally contributed to the more favourable neurological course of the disease in initially asymptomatic patients. However, the comparable age at first non-neurological manifestations of aceruloplasminemia in both neurologically symptomatic and asymptomatic patients weakens this consideration. In addition, no clear genotype-phenotype correlations have been reported in aceruloplasminemia [16]. Recent observations that suggest a milder phenotypic expression of mutations that allow the synthesis of apoceruloplasmin with some residual ferroxidase activity, need validation in larger series [17].

## Conclusions

Neurological outcomes of treatment for aceruloplasminemia, in both our three cases and other published cases, support the potential of iron chelation therapy to slow neurological progression when treatment is initiated in an early stage of the disease. Our analysis of aggregated case reports, which is the first of its kind in aceruloplasminemia, further highlights that reported outcomes of treatment in individual cases should be cautiously used to select the optimal therapeutic approach for aceruloplasminemia, given the possibly misleading contribution of various types of bias and often unsatisfying duration of follow-up. It underlines the need for further prospective studies using agreed treatment regimen with long-term follow-up. Based on the observed long-term safety and tolerability in our study and its theoretical superiority, combined iron chelation therapy with deferiprone and phlebotomy is recommended for patients without symptomatic anemia. In the presence of symptomatic anemia and decreased tolerability of phlebotomy, deferiprone with deferoxamine or deferiprone combined with another iron chelating method might be a reasonable alternative approach to achieve efficient iron chelation in aceruloplasminemia.

## Supplementary information

**Additional file 1.** Detailed overview of biochemical parameters of the G631R homozygous cases at baseline and during follow-up.

**Additional file 2.** Qualitative assessment of brain iron by T2-weighted MRI at 1.5 T in case 1.

**Additional file 3.** Detailed overview of published treatments for aceruloplasminemia.

## Data Availability

All data generated or analysed during this study are included in the published article and its supplementary information files.

## Abbreviations

*CP*: Ceruloplasmin
*HADS*: Hospital Anxiety and Depression Scale
NBIA: Neurodegeneration with brain iron accumulation
PKAN: Pantothenate kinase-associated neurodegeneration
*SARA*: Scale for the Assessment and Rating of Ataxia
*UPDRS*: Unified Parkinson’s Disease Rating Scale
