# Supplementary material for: Effects of iron chelation therapy on the clinical course of aceruloplasminemia: an analysis of aggregated case reports
Source: Orphanet J Rare Dis. 2020 Apr 25;15:105. doi: 10.1186/s13023-020-01385-w (PMC7183696; doi:10.1186/s13023-020-01385-w)
Supplement: Supplementary file 1 — Additional file 1. Detailed overview of biochemical parameters of the G631R homozygous cases at baseline and during follow-up. [file 13023_2020_1385_MOESM1_ESM.docx]

| **Additional file 1. Detailed overview of biochemical parameters of the G631R homozygous cases at baseline and during follow-up.** | | | | | | | | | |
| --- | --- | --- | --- | --- | --- | --- | --- | --- | --- |
| **Case 1** | Parameter | Unit |  | Follow-up in months | | | | | |
|  |  |  | *N* | 0 | 3 | 6 | 9 | 12 | 18^a^ |
|  | Hb | g/dl | *13.6-17.7* | 13.2 | 12.7 | 13.1 | 12.7 | 12.2 | 10.8 |
|  | Ht | l/l | *0.4-0.5* | 0.40 | 0.39 | 0.41 | 0.41 | 0.40 | 0.37 |
|  | MCV | fl | *80-100* | 91 | 88 | 87 | 83 | 73 | 65 |
|  | Reticulocytes | ·10^9^/l | *30-95* | 85.1 | *NA* | 64.5 | *NA* | 38.2 | *NA* |
|  | Neutrophils range | ·10^9^/l | *1.4-8.0* | 6.4 | 5.4-7.5 | 4.7-7.4 | 5.0-5.6 | 4.3-6.8 | 6.8-7.1 |
|  | Iron | µg/dl | *55.8-167.5* | 162.5 | 149.1 | 164.7 | 50.3 | 26.2 | 17.9 |
|  | Ferritin | ng/ml | *30-240* | 2131 | 1184 | 688 | 246 | 124 | 113 |
|  | Transferrin | mg/dl | *200-350* | 300 | 290 | 330 | 360 | 360 | 350 |
|  | TfS | % | *25-45* | 38 | 37 | 35 | 23 | 5 | 4 |
|  | Creatinine | µmol/l | *65-115* | 69 | 62 | 74 | 61 | 62 | 74 |
|  | Urea | mmol/l | *2.5-7.5* | 3.7 | 3.2 | 4.6 | 4.5 | 6.7 | 7 |
|  | AST | U/l | *0-34* | 39 | 27 | 36 | 23 | 15 | 12 |
|  | ALT | U/l | *0-44* | 56 | 38 | 41 | 25 | 23 | 15 |
|  | CRP | mg/l | *0-9* | 0.7 | 1.9 | 0.4 | 0.3 | 2.1 | 20 |
|  | Zinc | µmol/l | *64.3-124* | 90.1 | *NA* | *NA* | 96.7 | 107.8 | 12.1^b^ |
|  | Testosterone | nmol/l | *10-30* | 13.3 | *NA* | 8.2 | *NA* | 7.7 | *NA* |
|  | HbA1c | mmol/mol | *26-42* | 54 | *NA* | 42 | 50 | 70 | 61 |

Abbreviations: TfS - transferrin saturation; N - normal range; NA - not available.

^a^Patient died after 18 months of follow-up. Phlebotomy was stopped after 15 months and deferiprone after 17 months of treatment. Due to nausea and vomiting, deferiprone had been temporarily discontinued for 5 days after 5 months and 6 weeks after 12 months of treatment. ^b^Asymptomatic zinc deficiency, for which supplementation was initiated.

| **Case 2** | Parameter | Unit |  | Follow-up in months | | | | | | | | | | |
| --- | --- | --- | --- | --- | --- | --- | --- | --- | --- | --- | --- | --- | --- | --- |
|  |  |  | *N* | 0 | 3 | 6 | 9 | 12 | 18 | 24 | 30 | 36 | 42 | 48^a^ |
|  | Hb | g/dl | *13.6-17.7* | 12.7 | 13.1 | 13.1 | 11.3 | 13.1 | 11.6 | 13.4 | 12.6 | 12.9 | *NA* | 15.5 |
|  | Ht | l/l | *0.4-0.5* | 0.38 | 0.39 | 0.41 | 0.36 | 0.40 | 0.37 | 0.40 | 0.40 | 0.39 | *NA* | 0.48 |
|  | MCV | fl | *80-100* | 82 | 83 | 83 | 80 | 77 | 76 | 80 | 79 | 78 | *NA* | 83 |
|  | Reticulocytes | ·10^9^/l | *30-95* | 46.1 | 44.1 | *NA* | *NA* | 44.2 | 38.3 | 65.4 | *NA* | 58.9 | *NA* | 64.4 |
|  | Neutrophils range | ·10^9^/l | *1.4-8.0* | 6.8 | 3.7-6.9 | 1.4-3.1 ^b^ | 3.1-4.8 | 2.2-2.9 | 1.5-4.0 ^b^ | 3.0-4.5 | 3.6-3.7 | 2.9-6.4 | 5.1-5.2 | 5.3-10.1 |
|  | Iron | µg/dl | *55.8-167.5* | 63.1 | 111.1 | 67 | 64.2 | 76.5 | 40.2 | 80.4 | 67.0 | 37.3 | *NA* | 65.88 |
|  | Ferritin | ng/ml | *30-240* | 2164 | 1865 | 1752 | 1179 | 1181 | 985 | 839 | 605 | 505 | *NA* | 583 |
|  | Transferrin | mg/dl | *200-350* | 270 | 260 | 250 | 260 | 260 | 270 | 260 | 270 | 270 | *NA* | 260 |
|  | TfS | % | *25-45* | 17 | 30 | 19 | 18 | 21 | 12 | 22 | 18 | 9 | *NA* | 18 |
|  | Creatinine | µmol/l | *65-115* | 57 | 58 | 59 | 61 | 77 | 64 | 57 | 65 | 68 | *NA* | 60 |
|  | Urea | mmol/l | *2.5-7.5* | 6.5 | 6.8 | 4.7 | 5.6 | 10.2 | 4.7 | 5.4 | 6.3 | 5.9 | *NA* | 4.3 |
|  | AST | U/l | *0-34* | 24 | 31 | 28 | 21 | 14 | 14 | 26 | 16 | 15 | 12 | 13 |
|  | ALT | U/l | *0-44* | 42 | 42 | 47 | 39 | 29 | 22 | 31 | 32 | 26 | 27 | 27 |
|  | CRP | mg/l | *0-9* | 15 | 7.6 | 8.6 | 17 | 11 | 12 | *NA* | 7.3 | 16 | *NA* | 8.4 |
|  | Zinc | µmol/l | *64.3-124* | 92.7 | *NA* | *NA* | *NA* | 97.5 | *NA* | 93.6 | NA | 96.1 | 114.0 | 101.3 |
|  | Testosterone | nmol/l | *10-30* | 12.4 | *NA* | 17 | *NA* | 15.3 | *NA* | 7.2 ^c^ | 10.7 | 8.0 | 7.7 | 18.7 |
|  | HbA1c | mmol/mol | *26-42* | 87 | 73 | 63 | 62 | 62 | 66 | 56 | 68 | 65 | 58 | 58 |

Abbreviations: TfS - transferrin saturation; N - normal range; NA - not available.

^a^Total follow-up of 76 months; after 48 months of follow-up no further laboratory examinations were performed due to neurological deterioration. Phlebotomy was stopped after 34 months and deferiprone after 52 months of treatment. ^b^Deferiprone dose reduction due to a significant decrease in neutrophil count. ^c^ Testosterone supplementation was started and increased after 36 months of treatment because of persistent low testosterone levels.

| **Case 3** | Parameter | Unit |  | Follow-up in months | | | | | | | | | |
| --- | --- | --- | --- | --- | --- | --- | --- | --- | --- | --- | --- | --- | --- |
|  |  |  | *N* | 0 | 3 | 6 | 15 | 21 | 30 | 42 | 45 | 51 | 57^a^ |
|  | Hb | g/dl | *13.6-17.7* | 11.6 | 10.2 | 10.8 | 10.3^b^ | 11.1 | 12.9 | 13.2 | 12.7 | 12.9 | 13.1 |
|  | Ht | l/l | *0.4-0.5* | *NA* | *NA* | 0.35 | 0.35 | *NA* | 0.4 | 0.41 | 0.39 | 0.41 | *NA* |
|  | MCV | fl | *80-100* | 79 | 72 | 74 | 73 | 74 | 84 | 82 | 83 | 87 | 84 |
|  | Reticulocytes | ·10^9^/l | *30-95* | 37.7 | 30.0 | 34.2 | 52.4 | *NA* | 50 | 49.1 | 47.6 | 48.2 | 57.1 |
|  | Neutrophils range | ·10^9^/l | *1.4-8.0* | 2.9 | 2.4-4.5 | 2.2-2.7 | 2.2-3.7 | 3.9-4.3 | 2.4-3.3 | 1.5-3.6 | 2.5-3.5 | 3.2-3.5 | 2.7-5.4 |
|  | Iron | µg/dl | *55.8-167.5* | 31.8 | 21.2 | 31.3 | 26.2 | 24.6 | 43.0 | 43.6 | 46.3 | 34.6 | 72.0 |
|  | Ferritin | ng/ml | *30-240* | 266 | 158 | 152 | 111 | 133 | 253 | 911^c^ | 574 | 302 | 136 |
|  | Transferrin | mg/dl | *200-350* | 370 | 400 | 420 | 410 | 370 | 360 | 350 | 340 | 350 | 400 |
|  | TfS | % | *25-45* | 6 | 4 | 5 | 5 | 5 | 8 | 9 | 10 | 7 | 13 |
|  | Creatinine | µmol/l | *65-115* | 68 | 67 | 75 | 65 | 70 | 69 | 95 | 71 | 73 | *NA* |
|  | Urea | mmol/l | *2.5-7.5* | 5.1 | 6.3 | 4.4 | 6.7 | *NA* | 5.0 | 6.3 | 5.6 | 6.5 | *NA* |
|  | AST | U/l | *0-34* | 40 | 38 | 43 | 32 | 39 | 40 | 74 | 57 | 57 | 33 |
|  | ALT | U/l | *0-44* | 58 | 28 | 38 | 35 | 39 | 40 | 84 | 64 | 71 | 46 |
|  | CRP | mg/l | *0-9* | 0.3 | 1.2 | 0.6 | 0.4 | *NA* | 0.3 | 0.4 | 0.3 | 0.5 | *NA* |
|  | Zinc | µmol/l | *64.3-124* | 82.7 | 72.9 | 82.3 | 84 | 94.6 | 91.9 | 95.7 | *NA* | 84.3 | *NA* |
|  | Testosterone | nmol/l | *10-30* | 11.3 | 10.0 | 10.9 | 11.6 | 12.4 | 12.6 | 10.0 | *NA* | 8.26 | 11.57 |
|  | HbA1c | mmol/mol | *26-42* | 43 | 48 | 43 | *NA* | 44 | 40 | *NA* | *NA* | 49 | *NA* |

Abbreviations: TfS - transferrin saturation; N - normal range; NA - not available.

^a^Total follow-up of 70 months; after 57 months of follow-up further appointments were arranged in a hospital closer to his home. Follow-up visits between 6-15 months and 30-42 months were canceled by the patient. ^b^Deferoxamine (1000mg, s.c., twice weekly) was discontinued due to persistent symptomatic anemia. ^c^Deferoxamine (1000mg, s.c., once weekly) was readministered.
