# Supplementary material for: Effects of iron chelation therapy on the clinical course of aceruloplasminemia: an analysis of aggregated case reports
Source: Orphanet J Rare Dis. 2020 Apr 25;15:105. doi: 10.1186/s13023-020-01385-w (PMC7183696; doi:10.1186/s13023-020-01385-w)
Supplement: Supplementary file 2 — Additional file 2. Qualitative assessment of brain iron by T2-weighted MRI at 1.5 T in case 1. [file 13023_2020_1385_MOESM2_ESM.docx]

**
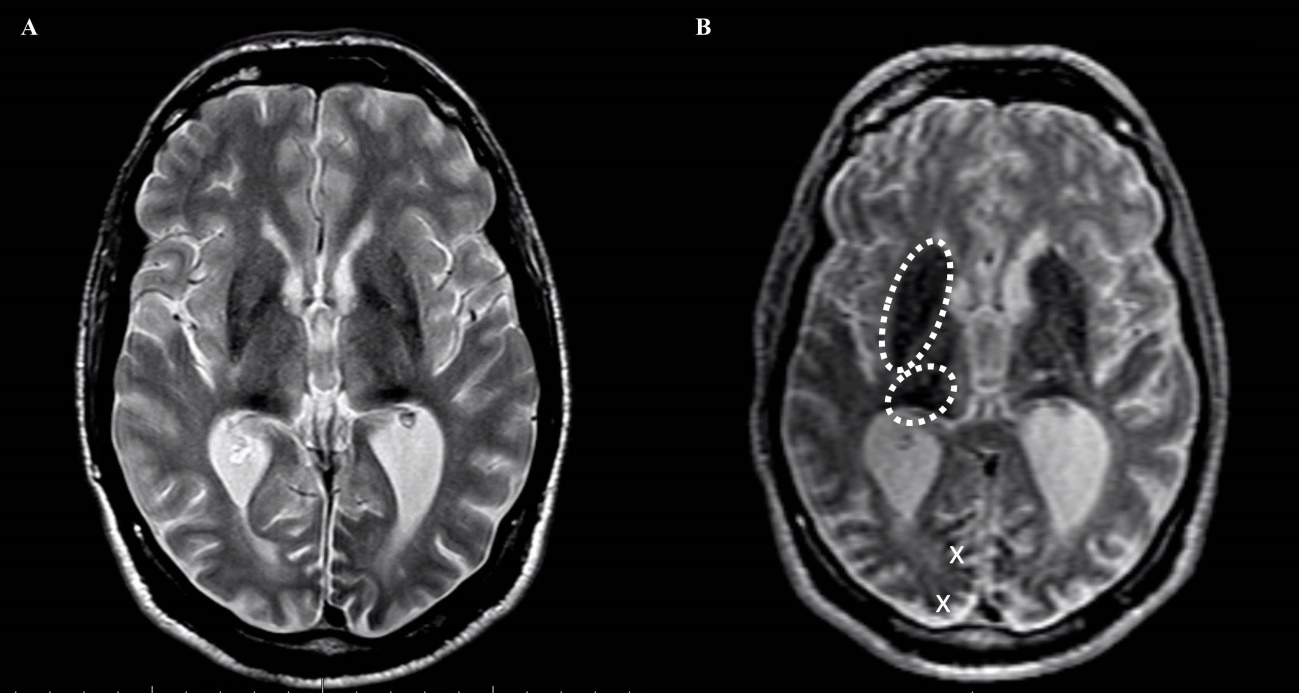
**

**Additional file 2.** **Qualitative assessment of brain iron by T2-weighted MRI at 1.5T in case 1.** Compared to baseline (A), progressive iron accumulation is suggested by more pronounced darkening of basal ganglia, pulvinar thalamus (dotted lines) and cerebral cortex (x) after 12 months of follow-up (B). Although comparable acquisition parameters were used, it should be noted that imaging was performed on different scanners. Comparison of T2*-weighted images was not possible, as this sequence was not included at baseline.
