# Supplementary material for: Effects of iron chelation therapy on the clinical course of aceruloplasminemia: an analysis of aggregated case reports
Source: Orphanet J Rare Dis. 2020 Apr 25;15:105. doi: 10.1186/s13023-020-01385-w (PMC7183696; doi:10.1186/s13023-020-01385-w)
Supplement: Supplementary file 3 — Additional file 3. Detailed overview of published treatments for aceruloplasminemia. [file 13023_2020_1385_MOESM3_ESM.docx]

| **Additional file 3. Detailed overview of published treatments for aceruloplasminemia.** | | | | | | | |
| --- | --- | --- | --- | --- | --- | --- | --- |
|  |  | Age at initiation of treatment, y | Duration of neurological symptoms, y | Duration of treatment | Neurological outcomes  *(Rating scales before – after treatment)* | Age at end of follow-up, y (ongoing follow-up) | |
| **Deferoxamine**  Neurologically symptomatic  - Miyajima et al. (1987, 1997), Kaneko et al. (2002)  - Morita et al. (1995), Kaneko et al. (2012)^a^  - Loréal et al. (2002)^b^ **+** **vitamin C**  - Haemers et al. (2004), Brugger et al. (2016)^a^  - Shang et al. (2006), Pan et al. (2011)  - Hida et al. (2010)^a^  - Ogimoto et al. (2011)  Asymptomatic  - Pérez-Aguilar (2005)  - Ogimoto et al. (2011) | **Daily dose**  500 mg 2d/w  NA  NA  2000 mg 5d/w  2400 mg 5d/w  500 mg 1d/w  1000 mg 1d/w  NA  NA  NA | 61  51  41  62  59  48  58  52  39  56 | 10  1  3  3  2  2  7  3  -  - | 10 m  NA  NA  1 y^c^  6 m^c^  4 y  4 y  NA  NA  NA | Improvement of blepharospasm, grimacing and rigidity; unchanged dysarthria  Progressive movement disorder  NA  NA  Stable blepharospasm and oromandibular dystonia  Progressive movement disorder and cognitive decline *(F-M 1 – 2; UPDRS/III 2 – 10; BARS 0 – 1)*  Improved movement disorder and stable cognitive decline after 1.5 years of treatment, followed by progressive decline  NA  NA  NA | 66  NA  56  63  59  52  62  NA  NA  NA | (†)  (†)  (†)  (NA)  (NA)  (NA)  (†)  (NA)  (NA)  (NA) |
| **Deferasirox**  Neurologically symptomatic  - Finkenstedt et al. (2010)^a^  - Roberti et al. (2011)  - Suzuki et al. (2013)  - Matsushima et al. (2014)^b^  - Riboldi et al. (2018)  Asymptomatic  - Finkenstedt et al. (2010)^a^  - Roberti et al. (2011)  - Rusticeanu et al. (2012)^b^  - Tai et al. (2013)  - Doyle et al. (2015)^b^  - Lindner et al. (2015)^b^ | **Daily dose**  15-20 mg/kg  17-20 mg/kg  10-20 mg/kg  500 mg  500 mg  NA  17-20 mg/kg  10-20 mg/kg  10 mg/kg  500-1000 mg  NA  4-15 mg/kg  4-15 mg/kg  4-15 mg/kg | 47  39  57  59  63  66  33  61  39  35  20  43  39  24 | 4  0  1  6  3  5  -  -  -  -  -  -  -  - | 3 m^c^  5 m^c^  16 m  4 m^c^  32 m  1 m  1 w^c^  9 m  7 y^c^  2 y  18 m  10 y  10 y  10 y | Stable cerebellar syndrome  Progression of movement disorder and mild cognitive decline  Improved psychotic symptoms  Improved movement disorder after 10 days, stable cognitive decline  Progressive movement disorder, cognitive decline, apathy *(SARA 7 – 14.5; MMSE 26/30 – 16/30)*  No significant improvements  Asymptomatic  Asymptomatic  Asymptomatic  Asymptomatic  Asymptomatic  Asymptomatic  Asymptomatic  Asymptomatic | 51  43  58  59  70  66  37  61  47  37  22  54  50  35 | (NA)  (NA)  (NA)  (†)  (yes)  (†)  (NA)  (NA)  (yes)  (NA)  (NA)  (yes)  (yes)  (yes) |
| **Deferiprone + vitamin C and E**  Neurologically symptomatic  - Pelucchi et al. (2018)  Asymptomatic  - Pelucchi et al. (2018) | **Daily dose**  35 mg/kg 3d/w  24 mg/kg 3d/w | 40  37 | 0  - | 11 y, 8 m  11.5 y | Development of behavioral changes, cognitive decline and parkinsonism  Development of neurotic disorder | 52  49 | (NA)  (NA) |
| **Phlebotomy**  Asymptomatic  - Hellman et al. (2000)  - Bjork et al. (2015)  - Watanabe et al. (2018) | **Frequency**  250 ml/w  NA-1x/4 m  1x/few m | 49  ~35  61 | -  -  - | 6 y  15 y ^c^  6 y | Development of mild movement disorder  Progressive movement disorder, cognitive decline and psychiatric changes  Development of movement disorder and cognitive decline | 55  ~50  67 | (NA)  (NA)  † |
| **Phlebotomy + deferasirox**  Asymptomatic  - Pelucchi et al. (2018) | **Frequency + daily dose**  400 ml/1-2 m (25x) + 12 mg/kg 4d/w | 53 | - | 51 m | Asymptomatic | 57 | (NA) |
| **Phlebotomy + deferiprone**  Neurologically symptomatic  - This study – case 1  - This study – case 2 | **Frequency + daily dose**  300-500 ml/2-4 w + 2000-4000 mg  300-500 ml/3-6 w + 2000-4000 mg | 50  56 | 2  2 | 5 m phb  15 m phb+DFP ^c^  4 m phb  34 m phb+DFP | Progressive movement disorder and cognitive decline *(UPDRS/III 20 – 28; SARA 12.5 – 23; HADS 6 – 12)*  Progressive movement disorder and cognitive decline *(UPDRS/III 6.5 – 31; SARA 9.5 – 27.5; HADS 13 – 6)* | 52  63 | (†)  (yes) |
| **Deferiprone + deferoxamine**  Asymptomatic  - Badat et al. (2015) | **Daily dose**  7 mg/kg 3-4d/w - 80mg/kg + 45 mg/kg 2d/w | 28 | - | 5 y, 3 m DFP  9 m DFP+DFO | Asymptomatic | 34 | (NA) |
| **Fresh frozen plasma**  Neurologically symptomatic  - Logan et al. (1994) | **Frequency**  300 ml/NA | 58 | 6 | NA | NA | NA | (NA) |
| **Fresh frozen plasma + deferoxamine/deferiprone**  Neurologically symptomatic  - Yonekawa et al. (1999), Kaneko et al. (2002)  - Poli et al. (2017)^b^ | **Frequency + daily dose**  450 ml 1d/w +  1000 mg / -  500 ml 1d/w + 1000 mg / 500mg | 54  59 | 5  0 | 6 w FFP  6 w FFP+DFO  5 d FFP+DFO  6 m FFP+DFP | Improved movement disorder  Improved movement disorder *(UPDRS/III 38 – 19; MMSE 18/30 – 19/30)* | 56  59 | (†)  (NA) |
| **Fresh frozen plasma + zinc**  Neurologically symptomatic  - Hines et al. (2018) | **Frequency + daily dose**  NA  NA | 54  51 | 1  0 | 4 y  4 y | Subjectively improved peripheral neuropathy  Subjectively improved gait disturbance and cognitive function | 58  55 | (NA)  (NA) |
| **Different consecutive treatments** | | | | | | | |
|  |  | Age at initiation of first treatment, y | Duration of neurological disease , y | Duration of treatment | Neurological outcomes | Age at end of follow-up, y (ongoing follow-up) | |
| **Deferoxamine – deferiprone**  Asymptomatic  - Pelucchi et al. (2018) **+ vitamin E**  - Mariani et al. (2004), Pelucchi et al. (2018) **+ vitamin C and E**  - Fasano et al. (2008), Bove et al. (2015)^b^ | **Daily dose**  10 mg/kg 3-4d/w – 35 mg/kg 3d/w  20 mg/kg 5d/w -  75 mg/kg; 35mg/kg 3d/w  500 mg – 2500 mg 2-7d/w | 49  40  35 | -  -  - | NA  8 m - 6 m; NA  7 y (3 cycles) - 10 y | Asymptomatic  Development of movement disorder and mild cognitive decline  Asymptomatic | 60  56  62 | (NA)  (NA)  (yes) |
| **Deferoxamine – deferasirox**  Asymptomatic  **-** Calder et al. (2017) | **Daily dose**  NA | 44 | - | 8 y^c^ - 16 y ^c^ | Progressive movement disorder, cognitive decline, psychiatric changes | 66 | (NA) |
| **Deferoxamine – deferasirox – deferiprone**  Asymptomatic  - Pelucchi et al. (2016, 2018) | **Daily dose**  1000 mg 3-4d/w - 13-19 mg/kg;  500 mg (6 mg/kg) – 67 mg/kg; 35 mg/kg | 40 | - | 5 y – 7 m; NA – 5 m^c^; NA^c^ | Development of cognitive decline, dysarthria | 52 | (NA) |
| **Deferoxamine – deferasirox – phlebotomy – deferiprone**  Neurologically symptomatic  - Bethlehem et al. (2010), Vroegindeweij et al. (2017) | **Daily dose -frequency**  NA – 500 mg – 1x/6w – 3000 mg | 59 | 8 | 4 m^c^ - 4.5 yr^c^ - 8 m^c^ - 3 m^c^ | Progressive movement disorder and cognitive decline | 65 | (†) |
| **Deferoxamine – deferiprone + phlebotomy – deferoxamine + deferiprone**  Asymptomatic  - This study – case 3 | **Daily dose - frequency**  1000 mg 2d/w - 300 ml/2w + 1000 mg - 2000-3000 mg + 1000 mg 1-2d/w | 48 | - | 13 y – 1 m^c^ - 43m^c^ | Development of gait disturbances, psychiatric changes *(UPDRS 2 – 9; SARA 0 – 4 with deferiprone + phlebotomy and deferoxamine + deferiprone)* | 67 | (yes) |
| **Deferoxamine – minocycline**  Asymptomatic  - Hayashida et al. (2016) | **Daily dose**  NA- 150 mg | 33 | - | 6 m^c^ - 1 y | Development of movement disorder and cognitive function that improved with minocycline *(UPDRS/III 32 – 20; ICARS 62 – 27; BPRS 80 – 26 with minocycline)* | 47 | (NA) |
| **Deferasirox – deferiprone + fresh frozen plasma**  Neurologically symptomatic  - Skidmore et al. (2008)^a^ | **Daily dose - frequency**  1000 mg - NA | 54 | 5 | 6 m – 2w | Improved movement disorder and cognitive decline *(MMSE 29/30 – 29/30; crossed response inhibition task 5/10 errors – 0/10 errors)* | 54 | (NA) |
| **Deferasirox – phlebotomy**  Asymptomatic  - Vroegindeweij et al. (2017) | **Daily dose - frequency**  250 mg - NA | 62 | - | 4 y – 2y | Asymptomatic | 69 | (NA) |

Abbreviations: d – day(s); w – week(s); m – month(s); yr– year(s); NA – not available; phb – phlebotomy; DFP – deferiprone; DFO – deferoxamine; DFX – deferasirox; FFP – fresh frozen plasma, F-M – Fahn-Marsden dystonia rating scale; UPDRS/III – unified Parkinson’s disease rating scale; BARS – brief ataxia rating scale; SARA – scale for the assessment and rating of ataxia; ICARS – international cooperative ataxia rating scale; MMSE – mini-mental state examination; HADS – hospital anxiety and depression scale; BPRS – brief psychiatry rating scale.

^a/b^Information was updated by direct contact with the authors in 2014/2019. ^c^(Temporary) discontinuation of treatment because of side effects. † Patient has died.
